# A narrative review of online food delivery in Australia: challenges and opportunities for public health nutrition policy

**DOI:** 10.1017/S1368980020000701

**Published:** 2020-06-09

**Authors:** Sarah Bates, Belinda Reeve, Helen Trevena

**Affiliations:** 1 School of Health and Related Research, Faculty of Medicine, Dentistry and Health, University of Sheffield, Sheffield S1 4DA, England; 2 Menzies Centre for Health Policy, Sydney School of Public Health, Charles Perkins Centre, University of Sydney, Sydney, New South Wales 2006, Australia; 3 The University of Sydney Law School, University of Sydney, Sydney, New South Wales 2006, Australia

**Keywords:** Online food delivery, Foodservice sector, Nutrition policy, Food environment

## Abstract

**Objective:**

Online food delivery (OFD) platforms offer consumers a convenient and fast delivery service of foods and drinks sourced from foodservice partners (e.g. restaurants, quick service restaurants). There is a need to assess the impact of this emergent segment of the foodservice sector on diet and diet-related health. The aim of this narrative review was to describe the OFD sector in Australia, its use and identify potential ways to include OFD platforms in existing public health nutrition policy.

**Design:**

A search was conducted in peer-reviewed and grey literature. Sources were analysed and synthesised to report the characteristics of OFD platforms, delivery process, users and potential drivers of usage. The aim and scope of public health nutrition policies were analysed to identify ways of including OFD platforms.

**Setting:**

Australia.

**Participants:**

General population.

**Results:**

There are three main operators with 9000–16 000 foodservice partners based predominantly in the main cities of Australia. OFD revenue has grown by 72 % in the last 5 years and is predicted to increase driven by usage by working adults with high disposable income who demand convenience. Current policies and initiatives aimed at manufacturers, retailers and foodservice outlets do not specifically regulate OFD platforms, although there is scope for these to be extended to such platforms.

**Conclusions:**

OFD platforms are disruptors of the foodservice sector. Innovative and consistent health policy options that target the unique challenges and opportunities posed by OFD platforms are required to limit the potentially negative impact of OFD platforms on diet and diet-related health.

Poor diet quality, specifically a diet low in fruits, vegetables and whole grains and high in foods containing added sugar, saturated fat and salt, is a key risk factor for non-communicable diseases (NCD)^(1)^ such as cardiovascular disease, some cancers and type 2 diabetes^(2)^. The food environment, defined as ‘the physical, economic, political and socio-cultural context in which consumers engage with the food system to make their decisions about acquiring, preparing and consuming food’^(3)^, is widely acknowledged as a driver of an increase in NCD in low-, middle- and high-income countries^(4)^. One aspect of the food environment is the foodservice sector. Traditionally, this sector includes dining and casual restaurants, cafes, and fast-food or quick service restaurants (hereafter, foodservice outlets). Collectively, these foodservice outlets provide foods and drinks prepared for immediate consumption either on- or off-premises^(5)^. Although there is limited research in Australia comparing the nutritional quality of these foods to those prepared in the home, there is evidence that eating takeaways regularly is associated with poorer diet quality^(6,7)^ and a higher prevalence of obesity^(7)^. Research based in the UK indicates that while home-cooked food might not be necessary for a high-quality diet^(8)^, many of these foods prepared away from the home are associated with having a high-energy, sugar, saturated fat and salt content^(9–13)^ and are less healthy than their home-cooked counterparts^(14,15)^.

A relatively new entrant to the foodservice sector in Australia is online food delivery (OFD) platforms (e.g. Ubereats). OFD platforms electronically connect consumers to a broad range of foodservice outlets. Consumers are presented with hundreds of menu choices to order online and request delivery at their convenience. Labelled ‘Food Delivery 2.0’, OFD platforms alter how we traditionally think about food providers^(16)^; they form part of the modern food environment in which smartphones are used to order food, access reviews and view pictures of meals^(17)^. OFD platforms can be described as ‘regulatory entrepreneurs’^(18)^, whereby they operate in a regulatory ‘grey zone’. In this situation, current public health nutrition policies are likely to be inapplicable, irrelevant, or OFD organisations know that new regulation can be resisted on the basis of hampering business growth^(18)^.

We suggest their presence raises concerns for public health nutrition. These concerns arise from the nutritional quality of the offering provided by foodservice outlets OFD platforms frequently partner with^(9–13)^, the promotion, availability and accessibility of unhealthy choices, and the association of these foods with diet-related NCD compared with meals prepared at home^(6,7,14,15)^. There are few published data in the public health nutrition literature describing OFD platforms – who owns them, how they work, who uses them, what customers order, when and why. Further, it is unknown to what extent existing public health nutrition policies incorporate OFD platforms. Australia currently lacks a coordinated national nutrition policy framework aimed at improving population nutrition. Although at a state level, there are menu labelling regulations in place^(19)^, at the federal level, initiatives that aim to improve population nutrition are voluntary and involve collaboration with the food industry^(20)^. However, excluding OFD platforms from these policy initiatives risks exacerbating an already uneven playing field and widening policy gaps. A greater understanding of OFD platforms and their potential to impact on the effectiveness of current public health nutrition policies is needed. The aim of this narrative review was to describe the OFD sector in Australia, its use, and to identify potential ways to include OFD platforms in public health nutrition policy.

## Methods

### Search criteria

We conducted a search of the academic literature using PubMed, Web of Science, Science Direct, Business Source Ultimate and ABI/Inform databases. The search strategy included terms related to ‘online food delivery’, ‘meal delivery’ or ‘takeaway’. Our search was supplemented with searches of the grey literature using Google, Australian Federal websites, Ibis World, Passport (by Euromonitor), WARC, company 360, capitol monitor and the Australian Food News website (www.ausfoodnews.com.au). The first ten pages (or equivalent to 100 results) were reviewed for relevant articles that were not identified in the database searches. We searched the reference lists of relevant papers to identify additional evidence sources. The search was restricted to sources available in English published between January 2009 and February 2019, as the focus was on recent developments in the foodservice sector involving OFD platforms operating in Australia. The search terms for each database and search results are given in supplementary file 1 (Table 2).

We included evidence sources that contained information about the characteristics (the OFD operators, how they operate and what they offer) and use (who uses OFD platforms, why and what is ordered) of OFD platforms in Australia. Given that OFD platforms in Australia have been notably present only in the last decade, we also included articles about OFD platforms and their foodservice partners from other high-income countries including the UK and USA. We did not include evidence sources that reported on the use of meal kits to be prepared at home or meal delivery programmes.

From the initial search, we identified the main OFD platform operators. In February 2019, we searched the websites of the three main OFD platforms in Australia for information about the OFD service and what it offered. For each OFD platform, this included the postcode areas they delivered to, the number of foodservice partners and the presence of quick service restaurants on the website. The Australian Government and State government websites and the WHO websites were searched for details of public health nutrition policies currently implemented in Australia (January–November 2019). We also examined best practice guidelines from four reports: Tackling NCD: ‘Best buys’ and other recommended interventions for the prevention and control of NCD^(21)^, World Cancer Research Fund NOURISHING framework^(22)^, The Healthy Food Environment Policy Index^(23)^ and the Heavy Burden of Obesity report^(24)^.

While one person conducted the search and led the analysis, all co-authors were involved through weekly meetings. All authors agreed the design of this study, discussed the approach to the analysis and debated early findings and competing interpretations.

### Analysis

Data reporting OFD platforms were analysed and synthesised to report the characteristics of OFD platforms, the OFD process, key users and potential drivers of OFD usage. Current public health nutrition policies implemented in Australia were identified through the government, state and WHO websites. The policy, its aim and scope and any current application to OFD platforms were analysed to identify relevance to OFD platforms to assess long-term impact. We categorised the recommendations listed in the four best practice guidelines (tackling NCD Best Buys^(21)^, World Cancer Research Fund NOURISHING framework^(22)^, The Healthy Food Environment Policy Index^(23)^ and the Heavy Burden of Obesity report^(24)^) that were relevant to OFD platforms into six domains: labelling, public awareness/mass media, reformulation, availability/portion size, fiscal and promotion/advertising (online Supplementary file, Table 1). We compared existing policies to these domains to determine which are addressed and where there are gaps in the current policy response that may impact on OFD platforms.

**Table 1:** Characteristics of online food delivery (OFD) platforms

	Delivery zone					
OFD platform	Major cities*	Other towns and regional area groupings	Own drivers	Launch year	Number of foodservice partners	National QSR chains†
Deliveroo	Adelaide, Brisbane, Cairns, Canberra, Geelong, Gold Coast, Melbourne, Perth, Sunshine Coast, Sydney, Wollongong		Yes	2014	~9000	KFC, Pizza Hut, Nandos, Oporto, Subway
Menulog	90 % of Australian addresses:		No	2006	~16 000	Hungry Jacks, KFC, McDonalds, Pizza Hut, Nandos, Oporto, Red Rooster, Subway, Zambrero
	Brisbane, Canberra, Gold Coast, Hobart, Melbourne, Perth, Sydney, Cairns, Darwin, Newcastle	Alice Springs, Byron Bay, regional New South Wales, Queensland (central coast, central western, north south eastern), Victoria (northern, south eastern, south western South Australia (far north, mid north, south, west coast, Hunter Valley region, Western Australia (south east, central, south western)			
Ubereats	Adelaide, Ballarat, Brisbane, Cairns, Canberra, Geelong, Gold Coast, Hobart, Melbourne, Newcastle, Perth, Sydney, Toowoomba, Townsville, Wollongong	Byron Bay	Yes	2016	~14 000	Dominos, KFC, McDonalds, Nandos, Oporto, Red Rooster, Subway

QSR, quick service restaurants.

* Major cities, populations of 100 000 people or more. Other towns, populations of <100 000^(31)^.

† Information taken from the OFD platform websites.

**Table 2:** Public health nutrition policies to improve the healthiness of foods and drinks for sale in Australian foodservice and retail outlets^(46)^

Policy	Overall description	Target of policy	Current application to OFD platforms in Australia and other countries	Dimensions from best practice guidelines	Relevance to OFD platforms and likely impact on diet-related health
Menu Kilojoule Labelling	A mandatory requirement to provide consumers with information on the energy content of their foods and drinks at the point of purchase. This scheme has been introduced by legislation in most Australian states (New South Wales, Victoria, Queensland, South Australia and the Australian Capital Territory)^(45)^. For example, in New South Wales (NSW), a foodservice operator of food outlets with twenty or more locations in NSW or fifty or more locations nationally must display the energy content of each standard food item in kilojoules (kJ). This includes ready-to-eat food, sold in single or multiple serves, standardised for portion size and content, shown on a menu or displayed with a piece or label. The restaurants must also display the recommended average daily kilojoule intake^(19)^. If the foodservice operator has their own app or website, they are required to display kilojoule content there as well^(19)^.	Foodservice outlets that meet requirements set out in the respective State legislation	Not applicable – In Australia, there is no guidance for (third party) OFD platforms. One OFD platform (Deliveroo) has voluntarily pledged to include energy information for up to 500 restaurants within the UK before implementing this internationally^(46)^.	Labelling	The technological feasibility is high for OFD platforms to display kJ information. OFD platforms already use promotional tagging to highlight different food types, offers and popularity, indicating it would highly feasible to add information that would aid healthy choices. There is evidence that menu labelling has prompted businesses to reformulate^(45)^, suggesting that the display of kJ information could lead to improvements in the nutritional quality of foods and drinks offered on OFD platforms. Deliveroo (which aims to include energy information in its app) is also working with a nutritionist and foodservice partners to introduce healthier options, which will be available through the app only^(46)^. The cost of the requirement to display the kilojoule information may disadvantage smaller foodservice partners currently excluded from the labelling legislation^(47)^.
Health Star Rating System (HSR)	A voluntary national labelling scheme, which aims to provide an easy way to compare similar packaged foods and encourage healthier choices^(48)^. A rating from 0·5 to 5 stars is generated for a food item based on energy, saturated fats, sodium and total sugar, fruit and vegetable, nut and legume content, dietary fibre and protein content. It is the food manufacturer and retailers’ responsibility to display the correct rating.	Packaged food	Not applicable	Labelling	There is evidence that the HSR can be applied to fast foods^(49)^ and therefore could be extended to meals offered on OFD platforms. Additional consideration may be needed for portion size, as the “per 100 g” method currently used may be more difficult for consumers to interpret that using standard portion sizes^(49)^. While it may require modifications to the HSR algorithm, it would also depend on the capacity of the foodservice partners to calculate the HSR and willingness of the OFD platforms to provide this information. A recent review indicates that the HSR ratings align well with the Australian dietary guidelines^(50)^, but the impact on consumer choice is less clear^(51,52)^.
Healthy Food Partnership	A partnership between the government, the public health sector and the food industry that aims to improve dietary habits by establishing a programme of voluntary product reformulation, encouraging healthier food choices, funding and implementing a national campaign promoting healthy eating and ultimately reducing the prevalence of overweight and obesity^(53)^.	Portion sizes, food choices	It is unclear whether OFD platforms have been approached about the partnership and opted not to be included or whether they have not been considered at all. Part of a planned foodservice pledge did include providing more nutritional information on app-based menu systems, but the working group responsible for this pledge had ceased operating^(54)^.	Reformulation, availability, mass media campaign/public awareness	Inclusion of OFD platforms in this partnership may enable a greater awareness and understanding of their impact on dietary quality and diet-related health for the government, public health sector and OFD platforms themselves. It could then facilitate discussions of how OFD platforms can be involved in encouraging healthier food choices that align with the goals of the partnership. However, there is limited evidence of the effectiveness of the Healthy Food Partnership^(55)^ or of a previously implemented initiative with similar goals^(56)^

## Results

### The online food delivery platforms in Australia

We found that, as of February 2019, there were three leading OFD platforms operating in Australia; Deliveroo (Deliveroo^®^, RooFoods Ltd)^(25)^, Uber eats (UberEATS^®^, Uber Technologies Inc.)^(26)^ and Menulog (Menulog^®^, Menulog Pty. Ltd)^(27)^ and Table 1 sets out their key characteristics. They each have between 9000 and 16 000 foodservice partners based predominantly in the main cities of Australia (e.g. Sydney, Melbourne, Perth, Brisbane and Canberra). Deliveroo and UberEATS employ delivery drivers to deliver meals from all foodservice partners, whereas partners of Menulog arrange their own delivery service. As well as partnering with foodservice outlets to fulfil orders prepared on-premise by the foodservice outlet, OFD platforms also provide commercial kitchens, known as ‘dark kitchens’, in which partners prepare the orders^(28)^. Another service, offered by Ubereats, is drop-in centres also known as ‘greenlight hubs’; these provide additional support for food delivery drivers such as advice on how to use the mobile phone application (‘app’)^(29)^.

Between 2014 and 2019, annual revenue growth for OFD platforms was 72 %^(30)^; this market outperformed the growth of the total foodservice sector which was 2·3 % over a similar period of time. However, total revenue was reported to be $278·1 (AUD) million which is approximately 0·5 % of the foodservice sector (valued at $53·9 billion in 2018). While growth of OFD platforms is expected to slow down, the predicted revenue increase is 15·4 % annually between 2019 and 2024 to $570·3 million^(30)^. This growth is being driven by an expansion of the scale or reach of OFD platforms from cities to regional towns and the services OFD platforms offer such as dark kitchens that provide a space for foodservice partners to prepare meals (for delivery only) and greenlight hubs^(28,29)^.

### Online food delivery process and key users of online food delivery platforms

**Fig. 1:**
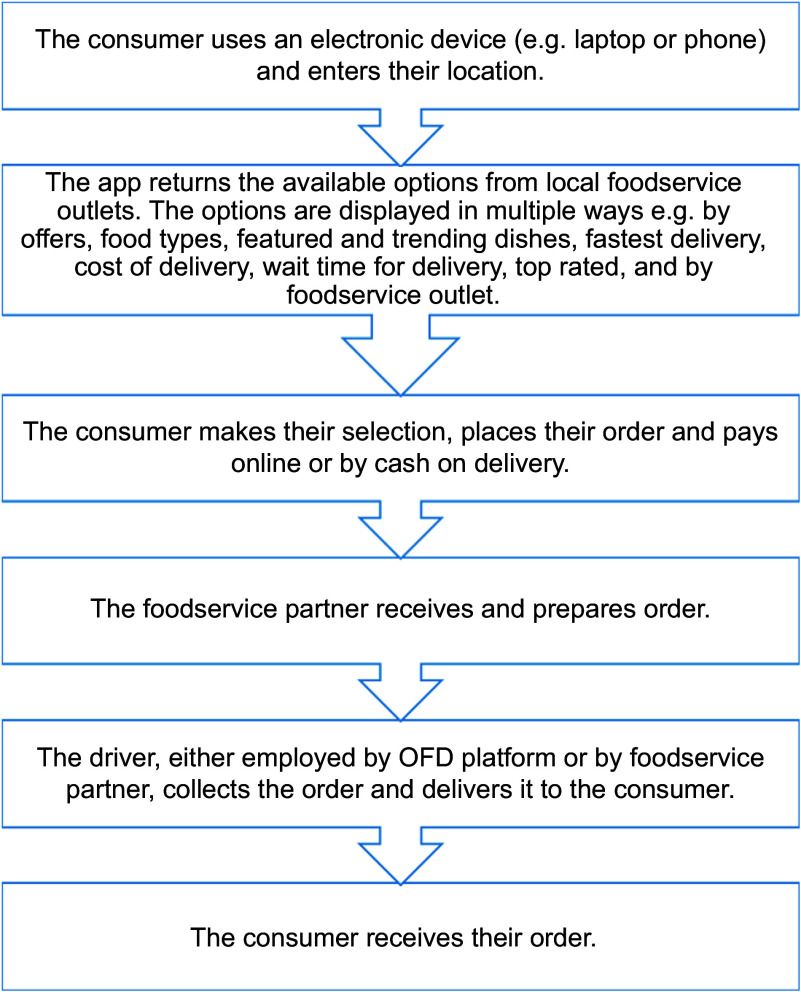
Online food delivery platform process

Figure 1 describes the OFD process – how consumers choose, order, pay for and take delivery of food and drink items using an OFD platform. Consumers have the option to use the OFD platform website or an app to browse menus from local foodservice outlets with the option of sorting and filtering by different characterises including offers, healthy options, cost of delivery and reviewer ratings^(25–27)^. The consumer places an order which is prepared by the foodservice outlet and then receives the order at the location of their choice.

One-third of Australian adults residing in cities used OFD platforms regularly, and the annual average spend per consumer was reported to be $1600 AUD in 2017^(32)^. According to market research reports, people using OFD platforms are typically working adults aged between 35 and 44 years with higher disposable incomes^(16,30,33,34)^. Foods are ordered for delivery at different locations including home and work. It was reported that meals ordered for delivery to workplaces are more likely to be unhealthy options^(35)^, although this was based on a report released by an OFD platform and so it is unclear how these orders were linked to the workplace or home and what was deemed healthy and unhealthy.

In a market research report, the most popular meal type (27·3 %) was reported to be ‘other’ – this category includes Mexican, Greek, vegetarian and salad-based meals. This shift away from traditionally popular takeaway options (Italian 23·8 %, Indian 15·6 %), and fast-food^(30,36)^ may reflect demand for foods perceived to be healthier^(30,37)^. However, although one OFD platform reported that ‘healthy’ food orders had increased by 1500 % across Australia in 2018^(36)^, it also found geographical variation in the types of food ordered and that fast-food remains the most popular option in some parts of Australia (e.g. Australian Capital Territory)^(35)^. Furthermore, although users have the option to select ‘healthy’ as a food category, we found no information on how OFD platforms categorise foods and drinks as ‘healthy’ and it is unclear if this promotional tag is applied by the foodservice partner or the OFD platform concerned.

### Potential drivers of online food delivery usage

The widespread adoption of Internet and mobile phone technology including the use of apps^(38)^ has enabled, and continues to drive, OFD usage. This technology underpins the OFD platforms’ proposition to meet consumer needs for convenience and choice. Convenience was reported to be a key driver in journal articles, news articles and market research reports and was said to reflect the busier lifestyles and long working hours of frequent users^(12,30,39–43)^. Consumer choice is another driver through access to premium options and multiple foodservice outlets through one app compared with traditional foodservice food outlets that can only provide a limited offering^(25–32,32–37)^.

### The Australian policy context and potential methods to include online food delivery platforms

We found no national public health nutrition policies (defined as ‘A broad statement of goals, objectives and a way to create a framework for policy action’^(44)^), targeting OFD platforms specifically either in Australia or internationally. Table 2 summarises the public health nutrition policies found^(53)^ along with details of the aim and scope, application to policy domain drawn from best practice guidelines, any current application to OFD platforms, and ways in which the policies could be extended to be relevant to OFD platforms.

The Federal Government has implemented initiatives in many of the policy domains recommended by Global authorities. These include some labelling (including front and back of pack, and menu labelling), mass media campaigns, reformulation and availability/portion size. However, the initiatives are voluntary, and there is little or mixed evidence to support their effectiveness in any of these domains and there were no initiatives that explicitly targeted promotion/adverting or that used fiscal measures.

We found three policies^(53)^ relevant to OFD platforms: one state-based initiative, Menu Kilojoule Labelling^(19)^, and two policy initiatives, the Health Star Rating (HSR) System^(44)^ and the Healthy Food Partnership^(54)^. Menu Kilojoule Labelling, depending on state-specific legislation, requires certain foodservice outlets to provide consumers with information on the energy content of their food and drinks at the point of purchase. This impacts on some foodservice partners of OFD platforms but does not apply to OFD platforms specifically. The HSR and the Healthy Food Partnership are both voluntary policies. The HSR system enables manufacturers and retailers of food to provide a rating from 0·5 to 5 stars based on nutritional components on packaged foods, and research suggests that this system could also be applied to fast food^(49)^. The Healthy Food Partnership^(54)^ brings together the Federal Government, the public health sector and the food industry with the aim of tackling obesity. Although a plan to include more nutritional information on app-based menu systems was included as part of a foodservice pledge, the working group responsible for this pledge had ceased operating^(55)^. To the best of our knowledge, there is no evidence that these policies apply to, or target, OFD platforms specifically.

## Discussion

OFD platforms are a new addition to the foodservice sector and provide consumers with access to many foodservice partners. There are three main OFD platforms in Australia, each with a large number of foodservice partners. OFD platform revenue represents a small percentage of the overall foodservice sector but recently observed revenue growth is expected to increase. We found that these platforms are most commonly used by young working adults with higher disposable incomes and that there is evidence of a shift away from traditionally popular takeaway options such as fast food to a greater variety of food types. Convenience is the main driver of OFD platform use. An increase in the desire for convenience food is reported to be a result of rising household incomes, urbanisation and a reduction in the time available for activities such as cooking. More recently, a move towards urban living to access better education and employment opportunities has resulted in greater constraints on time and available cooking space^(57)^. More generally, reasons given for consuming meals prepared away from home include the desire for fast and filling meals, the cost and effort of cooking at home and a lack of time^(58–60)^. Whether these are drivers of OFD usage specifically remains unclear due to lack of available evidence.

OFD platforms are disruptors to the foodservice sector; they are altering the way that many of us choose and order takeaway meals and they present unique public health nutrition policy challenges. The availability of takeaway options has shifted from the physical to the virtual with hundreds of restaurants available at the tap of a screen^(61)^. In addition, OFD platforms have introduced an additional and new route to the market as they do not fit into the categories of foodservice outlet, food retail outlet or manufacturer. We believe the relative newness of OFD platforms at the time of this review was a factor in its absence from public health nutrition policies. As a new entrant with a novel business model, this is likely to add an additional challenge and complexity to national policymakers.

Examination of Global authority recommendations^(21–24)^ and translation into Australian policy initiatives showed that, although the initiatives address some of the best practice policy domains, only menu labelling is mandatory and this is based on state-specific legislation rather than a national scheme. We also found no evidence that public health nutrition policies explicitly target the advertising and promotion used by those in the food service sector. The HSR and Healthy Food Partnership address front of pack labelling, mass media campaigns, reformulation and availability/portion size but are voluntary and/or collaborative initiatives with the food industry rather than government-led regulation. Furthermore, a significant body of research suggests that initiatives predicated on voluntary action have limited success^(62)^. The unique OFD platform model and the relative absence of a robust national policy and policy process suggest that including OFD platforms in current initiatives will be challenging. Yet, the absence of policy to address OFD platforms enhances what many already consider to be an uneven playing field between suppliers of food^(63)^. For example, the lack of information available for smaller independent restaurants limits consumers’ ability to make an informed decision when choosing between several foodservice outlets with and without this information provided^(64)^.

We have identified three main ways worthy of further consideration. Consumers would be able to make more informed decisions if OFD platforms were required to provide the method and criteria used, if any, to label food options or foodservice partners as ‘healthy’. If not used already, this categorisation should be objective and based on nutritional content rather than a label selected by the foodservice partner. Adoption of nutritional information on the menus of foodservice partners would provide a transparent and objective categorisation method. A policy option that is likely to be highly feasible for OFD platforms is to incorporate nutrition information at least for foodservice partners who already provide this to comply with kilojoule menu labelling. The coupling of this nutritional information with the information already on the OFD platform is likely to be straightforward given that the foodservice partners will already have this information. It could also provide insights into how to best incorporate smaller foodservice partners as part of a phased approach to include all foodservice partners. Further, it would align with potential plans for a national approach to kilojoule labelling^(45)^. Second, although the HSR is currently targeted at packaged food, there is evidence that it can be applied to fast food^(49)^ indicating that it may be feasible to include HSR for food and drink options through OFD platforms. Research demonstrated that determining and displaying portion size was difficult^(49)^ and so implementation would be particularly dependent on the cooperation and collaboration of multiple foodservice partners. A third potential policy option is participation in government-led schemes such as the Healthy Food Partnership; this could provide an opportunity for meaningful dialogue between stakeholders (e.g. OFD platforms, the government and the public health sector) to increase the collective understanding of how OFD platforms could make a positive impact on diet and health outcomes and implement actions that align with the goals of the partnership. However, as the foodservice working group has ceased operating, it is difficult to determine what actions will be implemented and therefore how OFD platforms can be included in this initiative.

Public health nutrition policymaking has been shown to be a hotly contested arena with multiple vested interests^(20)^. The recent emergence and growth of OFD platforms add to the current challenges faced by policymakers. OFD platforms operate in a policy and legal ‘grey zone’, and there are similarities between OFD platforms and other regulatory entrepreneurs. For example, Uber resisted being labelled as a taxi service, which enables them to operate outside of the regulation associated with this classification^(65)^. Likewise, it is unclear if OFD platforms are categorised as manufacturer, retailer or foodservice outlet, and therefore they fall outside the policies we identified as targeting these types of organisations. OFD platforms also make use of technological advancements that may fall outside current and future regulation. In the US, ground robots have been used to make commercial deliveries on university campuses and trials of autonomous vehicles and drones to deliver orders are ongoing^(66–68)^. Other technologies such as voice recognition could further increase the convenience of OFD services^(66)^. Like Uber, the OFD industry and main operators within it have also become so large that regulation restricting use in any way, particularly increased costs or reduced convenience, would likely be opposed by OFD platform organisations and the large number of consumers that use and support these services^(18)^. In order to design and implement effective policies, policymakers must be able to at least match the pace of development set by OFD platforms.

OFD platforms could be well placed to positively impact the food environment through the application of technology, which underpins their business model, in areas of delivery and marketing. In relation to delivery, OFD could improve accessibility to healthier food. It is well established that areas of greatest disadvantage have a higher proportion of quick service restaurants and unhealthy takeaway options^(69)^; OFD platforms enable consumers to purchase from foodservice outlets outside of their immediate geographical location, potentially increasing the healthy options available to consumers in more disadvantaged areas. OFD platforms could also positively impact on population health through marketing and promotion. This is particularly important as young adults are key users of OFD platforms and growth in obesity is predicted to be higher in this group than in older ages^(70)^. This could be implemented using choice architecture; a method of changing the environment to encourage or ‘nudge’ consumers towards healthier choices^(71)^, during the order process. For example, when presented with available menu options, choice architecture techniques including setting healthy items as defaults, restructuring the menu to highlight healthier options using methods such as promotional tagging, or recommending a healthier alternative to a previously ordered meal can all be implemented to encourage healthy options. These changes could be implemented with relative ease and, due to the high number of foodservice partners, would almost certainly have a larger impact than changes to individual foodservice partners. Adopting these strategies to increase healthy eating would require action from the OFD platforms themselves. It is argued in political theories of corporate social responsibility that companies should use their power and influence as a business in a socially responsible way^(72)^; in the context of OFD platforms and population health, this could include investigating and implementing methods to promote healthier food choices. However, although they may be motivated by evidence of consumers who are increasingly wanting healthier food choices^(73)^ and one platform has pledged to add nutrition information to all menus, there is little evidence that those in the food industry have taken meaningful action to promote a healthier food environment^(74)^. It is likely that policy inertia, opposition to policies by powerful commercial interests and lack of demand for policy action by the public, are powerful disincentives to meaningful change^(75)^.

In this review, we found no information about the marketing strategies used by individual OFD platform providers. However, we did find widespread use of promotional tagging to identify ‘healthy’ options. The technological capability of these platforms enables the collection of consumer purchase behaviour which could be used to inform targeted advertising campaigns through email and app notifications. While not unique to OFD platforms, these digital promotion techniques go beyond standard print and media to use a mix of social media channels, influencers and food bloggers^(76)^. Comprehensive research on, and identification of, digital marketing strategies already used would facilitate a comprehensive discussion on what policy options might tackle this aspect of the food environment.

There were some limitations of our review. Due to the paucity of published research at the time of this study, we included data from countries outside Australia (UK and USA) and thus some findings may not be as applicable to the Australian context. For example, while some OFD platforms aim to provide menu labelling for hundreds of restaurants in the UK (before extending this to all countries), there may be additional challenges when applying this to Australia due to the greater proportion of independent restaurants^(77,78)^. In addition, we found very few peer-reviewed papers related to OFD platforms. The majority of the information used to inform this narrative review was sourced from the grey literature, news articles, websites and market research reports. Although through our search strategy, we took care to only include sources from reputable organisations, the lack of peer review evidence could have resulted in some bias. We have highlighted throughout the review when information has been sourced from the OFD platforms themselves, as this could reflect marketing strategies rather than evidence-based data and should be viewed with caution. However, several academic databases were searched, and thus this highlighted the newness and lack of academic research on OFD platforms and the urgent need for this review and continuing research in this area.

## Conclusion

The popularity of OFD platforms is growing; they are receiving an increasing number of orders, offering more services and making use of advancing technology^(64,65)^. This creates additional challenges for public health nutrition policymakers. There is also the potential to channel the influence of OFD platforms to increase the number of healthy options available and to ‘nudge’ consumers towards these options, but this requires co-operation from the OFD platforms themselves which may be difficult to obtain. While anecdotal evidence suggests that OFD platforms are using unique forms of marketing to target consumers, there is currently little to no academic research on these strategies – a clear gap in the evidence base that needs to be addressed. OFD platforms are disruptors to the foodservice sector. The role of OFD platforms within the foodservice sector and the unique challenges and opportunities they pose should be considered when creating policies to improve public health nutrition and diet-related health outcomes.

## Supplementary material

For supplementary material accompanying this paper visit https://doi.org/10.1017/S1368980020000701. click here to view supplementary material
