# Supplementary material for: A narrative review of online food delivery in Australia: challenges and opportunities for public health nutrition policy
Source: Public Health Nutr. 2020 Jun 9;26(1):262–72. doi: 10.1017/S1368980020000701 (PMC7613985; doi:10.1017/S1368980020000701)
Supplement: Supplementary file 1 [file S1368980020000701sup001.docx]

Supplementary File 1

Table 1: Domains of Public health nutrition recommendations

| **Domains** | **Tackling NCDs Best buys** | **WCRF NOURSING** | **The Healthy Food Environment Policy Index (Food-EPI)** | **The heavy burden of obesity report** |
| --- | --- | --- | --- | --- |
| Labelling | - Reduce salt intake through front-of-pack labelling - Implement nutrition labelling to reduce total energy intake (kcal), sugars, sodium and fats | - Nutrition labels standards and regulations on the use of claims and implied claims of food | - Food labelling - health-related labelling for foods and beverages | - Food labelling - Menu labelling |
| Mass media campaign/public awareness | - Reduce salt intake through a behaviour change communication and mass media campaign - Implement mass media campaign on healthy diets, including social marketing | - Inform people about food and nutrition through public awareness |  | - Mass media campaigns |
| Reformulation | - Reduce salt intake through reformulation and setting target levels for the amount of salt in foods/meals - Replace trans-fats and saturated fats with unsaturated fats through reformulation, labelling, fiscal or agricultural policies | - Improve nutritional quality of the whole food supply - Harness food supply chain and actions across sectors to ensure coherence with health | - Food composition – nutrient composition of foods and non-alcoholic beverages |  |
| Availability/portion size | - Develop legislation to eliminate trans fats in food chain - Limiting portion and package size to reduce energy intake and risk of overweight/obesity | - Set incentives and rules to create a healthy retail and food service environment | - Food retail - availability of healthy and unhealthy foods |  |
| Fiscal | - Implement subsidies to increase consumption of fruit and vegetables | - Use economic tools to address food affordability and purchase incentives | - Food prices - relative price and affordability of ‘less healthy’ vs ‘healthy’ foods, meals and diets | - Price policies - Healthy food subsidies for health purposes - Changes in nutritional community environment through economic incentives |
| Advertising/promotion |  | - Restrict food advertising and other forms of commercial promotion | - Food promotion - exposure and power of promotion of unhealthy foods and beverages | - Regulation of advertising |
| Not relevant to OFD | - Reduce salt intake through supportive environment in public institutions (e.g. schools) to provide lower sodium options - Reduce sugar consumption through SSB tax - Promote and support breastfeeding in first 6 months of life - Implement nutrition education and counselling in different settings (e.g. schools) to increase intake of fruits and vegetables | - Offer healthy food and set standards in public institutions and other specific settings - Nutrition advice and counselling in health care settings - Give nutrition education and skills | - Food provision - nutritional quality of foods and beverages provided in different settings (e.g. schools) - Food trade and investment - the impacts of trade and investment agreements on the healthiness of food environments | - Prescription of physical activity by primary care doctors - School-based and other environmental policies that can influence children - Workplace policies - Policies promoting active transport and walking |

Table 2. Search terms used for number of results returned for each database.

| Database | Search terms | Total number of results |
| --- | --- | --- |
| Pubmed | (((((((online food delivery[Title]) OR meal delivery[Title]) OR takeaway[Title]) OR take-away[Title]))) AND "last 10 years"[PDat]) | 71 |
| Web of science | TITLE: ("online food delivery") OR TITLE: (meal AND delivery) OR TITLE: ("takeaway" AND food) OR TITLE: ("home deliver*" AND food) OR TITLE: ("take-away" AND food) OR TITLE: ("food delivery") Timespan: 2009-2019 | 223 |
| Science direct | (online food delivery OR (takeaway AND food) OR (take-away AND food) OR meal delivery) in title abstract and key words in 2009-2019 | 306 |
| Business source ultimate | TI online food delivery OR TI ( meal AND delivery ) OR TI ( takeaway AND (food OR meal) ) OR TI ( take-away AND (food OR meal) ) OR TI ( home delivery AND (food OR meal) ) | 97 |
| ABI/Inform | ti(online food delivery) OR ti(meal AND delivery) OR ti(takeaway AND (food OR meal)) OR ti(takeaway AND (food OR meal)) OR ti(take-away AND (food OR meal)) AND ti(home delivery AND (food OR meal) ) | 443 |
| Factivia | ti(online food delivery) OR ti(meal AND delivery) OR ti(takeaway AND (food OR meal)) OR ti(takeaway AND (food OR meal)) OR ti(take-away AND (food OR meal)) AND ti(home delivery AND (food OR meal) ) (Australia only | 177 |

Table 3: Details of evidence sources included in the review

| **First author** | **Year** | **Title** | **Type** | | **Topic/relevant information** |
| --- | --- | --- | --- | --- | --- |
| Australia and New Zealand Ministerial Forum on Food Regulation | 2018 | Review of fast food menu labelling schemes | Consultation paper | | Impact of labelling |
| Australian Food News (no author name) | 2018 | Five rising trends in the Australian foodservice market | News article | | Trend towards a healthy takeaway |
| Azoth Analytics | 2017 | Global online food delivery and takeaway market - Analysis by order type, by region by country: trends, opportunities and forecasts (2016-2021) | Market research report | | Market details about the food delivery and takeaway sector globally |
| Banner, A. | 2018 | Australians spend $1590 each year on delivered food | News article | | Use of OFD platforms |
| BIS Research | 2016 | Global Food Tech Market, by service type (online grocery delivery, online food aggregator & convenience services), by technology, by geography (North America, Asia-Pacific, Europe, & ROW) - Analysis & Forecast 2016-2022. | Market research report | | Ordering online market |
| Cain, J. | 2018 | Food delivery apps used by more than two million Australians aged 14+ according to Roy Morgan | News article | | Use of OFD platforms |
| Cain, J. | 2018 | What can we learn from Menulog’s online meal ordering figures? | News article | | Growth trends in Australian online food delivery market. |
| Commonwealth of Australia | 2014 | Health Star Rating System | Website | | Details of the health star rating system government scheme |
| Dunford, EK. | 2017 | A comparison of the Health Star Rating system when used for restaurant fast foods and packaged foods | Journal article | | Application of health star rating system to fast food |
| Euromonitor International | 2016 | Online, mobile and delivery: Three trends that are changing the way we dine out | Market research report | | Trends in the foodservice sector including desire for increased convenience |
| Farrell, S. | 2019 | Deliveroo to add takeaway food calorie counts | News article | | OFD platforms reports planned changes |
| Friend, E | 2016 | Online and Mobile Spending in Foodservice | News article | | Driver of OFD platform use |
| Hamlin, r. | 2016 | Does the Australasian “health star rating” front of pack nutritional label system work? | Journal article | | Effectiveness of health star rating system |
| Hogan, A. | 2017 | The evolution of Australia’s eating habits | News article | | Use of OFD platforms |
| Hughes N. | 2019 | Why we're still waiting for out-of home calories labelling | News article | | Reasons that kj labelling hasn’t been made mandatory at a federal level |
| Jones, A. | 2018 | Defining ‘unhealthy’: A systematic analysis of alignment between the Australian dietary guidelines and the Health Star Rating system | Journal article | | Matching of health star ratings to current dietary guidelines |
| Jones, A. | 2016 | Designing a healthy food partnership: Lessons from the Australian food and health dialogue | Journal article | | Guidelines for the healthy food partnership |
| Maganja, D. | 2019 | Consumer choice and the role of front-of-pack labelling: the Health Star Rating system | Journal article | | Effectiveness of health star rating system |
| Mitchell, S | 2019 | Coles and Uber Eats test ready-to-eat meal deliveries | News article | | Additional services offered by Ubereats |
| Nakos, N | 2017 | Will Deliveroo compete with its own customers? | News article | | Launch of ‘dark kitchens’ |
| O’Sullivan, S. | 2018 | Healthy fast food tickling nation's tastebuds | News article | | Types of foods ordered (reported by Deliveroo) |
| TechNavio | 2016 | Global Delivery and Takeaway Food Market 2016-2020 | Market research report | | Market details about the takeaway sector globally |
| Voung, B | 2018 | Online Food Ordering and Delivery Platforms in Australia | Market research report | | Market details about OFD platforms in Australia |
| World health organisation | 2019 | Policies in Australia | Website | | Details of currently implemented policies in Australia |
| **OFD platform websites** | | | | | |
| **OFD platform** | **Website** | | | **Year accessed** | |
| Deliveroo | https://deliveroo.com.au/ | | | 2019 | |
| Uber eats | https://www.ubereats.com/ | | | 2019 | |
| Menulog | https://www.menulog.com.au/ | | | 2019 | |
